# Allele coding in genomic evaluation

**DOI:** 10.1186/1297-9686-43-25

**Published:** 2011-06-26

**Authors:** Ismo Strandén, Ole F Christensen

**Affiliations:** 1 Biotechnology and Food Research, MTT Agrifood Research Finland, FI-31600 Jokioinen, Finland; 2 Aarhus University, Faculty of Agricultural Sciences, Dept of Genetics and Biotechnology, Blichers Allé 20, P.O. BOX 50, DK-8830, Tjele, Denmark

## Abstract

**Background:**

Genomic data are used in animal breeding to assist genetic evaluation. Several models to estimate genomic breeding values have been studied. In general, two approaches have been used. One approach estimates the marker effects first and then, genomic breeding values are obtained by summing marker effects. In the second approach, genomic breeding values are estimated directly using an equivalent model with a genomic relationship matrix. Allele coding is the method chosen to assign values to the regression coefficients in the statistical model. A common allele coding is zero for the homozygous genotype of the first allele, one for the heterozygote, and two for the homozygous genotype for the other allele. Another common allele coding changes these regression coefficients by subtracting a value from each marker such that the mean of regression coefficients is zero within each marker. We call this centered allele coding. This study considered effects of different allele coding methods on inference. Both marker-based and equivalent models were considered, and restricted maximum likelihood and Bayesian methods were used in inference.

**Results:**

Theoretical derivations showed that parameter estimates and estimated marker effects in marker-based models are the same irrespective of the allele coding, provided that the model has a fixed general mean. For the equivalent models, the same results hold, even though different allele coding methods lead to different genomic relationship matrices. Calculated genomic breeding values are independent of allele coding when the estimate of the general mean is included into the values. Reliabilities of estimated genomic breeding values calculated using elements of the inverse of the coefficient matrix depend on the allele coding because different allele coding methods imply different models. Finally, allele coding affects the mixing of Markov chain Monte Carlo algorithms, with the centered coding being the best.

**Conclusions:**

Different allele coding methods lead to the same inference in the marker-based and equivalent models when a fixed general mean is included in the model. However, reliabilities of genomic breeding values are affected by the allele coding method used. The centered coding has some numerical advantages when Markov chain Monte Carlo methods are used.

## Background

There has been growing interest in the use of marker-based models [1] in recent years. In studies using these models, descriptions of the effect of allele coding system on inference and computations are often vague or missing. By allele coding, we refer to the coefficients in the marker matrix of marker-based models. Coefficients, commonly used for allele coding of a marker is 0 when the individual is homozygous for the first allele, 1 when the individual is heterozygous, and 2 when the individual is homozygous for the second allele. Depending on which of the alleles has been chosen as the first allele, the coefficients are different. Thus, this allele coding method does not give unique regression coefficients.

There are other allele coding methods such as the one that use coefficients -1, 0, and 1 instead of 0, 1, and 2, respectively. Different allele coding methods affect coefficients in the statistical models but they do not seem to change the amount of information for statistical inference. Hence, one would expect that the use of different allele coding methods would lead to the same inference. However, allele coding can be of vital importance in computations. First, convergence of iterative methods such as Markov chain Monte Carlo (McMC) methods often used in Bayesian inference can be assumed to be affected by the allele coding method used because different allele coding methods change the correlation structure between marker effects. Second, equivalent models have become popular in animal breeding [2,3]. An important concept in these methods is the genomic relationship matrix. Differences in allele coding will yield different genomic relationship matrices [4]. Thus, some elements of the inverse of the coefficient matrix can be different and, consequently, reliabilities may be different.

We investigated effects of different allele coding methods using theoretical derivations and a practical example when restricted maximum likelihood (REML) or Bayesian inference is used. Effects on parameter estimates, reliabilities, and McMC computations were studied. We considered marker models and their equivalent breeding value models.

## Methods

### Genomic marker model

Let us consider a univariate linear mixed effects model for genomic marker effect estimation, e.g. [1],(1)

where **y **is *n *× 1 vector of observations, *μ *is the general mean, **Z **is a *n *× *m *matrix containing a column for each marker locus, **g **is a *m *× 1 vector of random SNP marker effects, and **e **is a random residual vector. There can be other fixed or random effects in the model but their inclusion does not change the following derivations.

### Allele coding methods

There are several alternatives for coding the coefficients in the **Z **matrix. Four allele coding systems are considered. A simple transformation can be made from one allele coding system to another. Our basic allele coding system counts the number of copies of one of the alleles. Depending on which of the alleles is counted, the matrix can be different. In the allele coding system 012, the number of copies of the less frequent allele is counted. Thus, the coefficient is 0 if the individual is homozygous for the more frequent allele, 1 if it is heterozygous, or 2 if it is homozygous for the less frequent allele. In this case, the **Z **matrix for the basic allele coding system 012 is denoted by **Z**_0_.

A general form for the allele coding transformation from the basic allele coding system is  where **v***_m _*is a *m *× 1 vector. This allows many types of allele coding methods. Note that the transformation keeps distances between allele codes within a marker the same. So, the coding 0,1,2 can be changed to - 1,0,1 or 0.5, 1.5, 2.5 by this transformation, but not to -10,0,10.

We define the centered allele coding system as **Z***_c _*= **Z**_0 _- **P***_c_*, where each column of the matrix **P***_c _*contains the average allele count for the corresponding marker column. Thus, summing values in each column will give a vector of zeros, i.e.,  is a vector of zeros. For the centered allele coding system, we have , i.e., . Note that **v***_m_/*2 gives the allele frequencies of the markers in the data.

The allele coding transformation allows shifts in the allele codes. The 101 allele coding system is such that - 1 is assigned to the genotype homozygous for the more frequent allele, 0 to the heterozygous individual, and 1 to the individual homozygous for the less frequent allele. For the 101 allele coding system, we have **v***_m _*= **1***_m_*. The 101 allele coding system is equal to the centered allele coding system when all allele frequencies are equal to 0.5.

In the following, the derivations will use the general allele coding transformation. The matrix **Z**_0 _is unique. However, in general the decision on which of the alleles to count is arbitrary. Let the 210 allele coding system be such that the more frequent allele is counted. Then the **Z **matrix for the 210 allele coding system can be calculated from the 012 coding matrix by  where **1***_n _*is *n *× 1 vector of ones. The 210 allele coding system is the opposite to the 012 allele coding system but results in this paper apply to the 210 allele coding system as well, with some modifications mentioned separately.

Different allele coding methods imply different models (1) and different models may lead to different parameter estimates. However, the inference considered in this paper is not affected by allele coding, as we demonstrate below.

### Inference in marker-based model

Variance component estimation by restricted maximum likelihood (REML), prediction of random effects, and Bayesian inference are all based on the likelihood after marginalization of the fixed effects, i.e., the fixed effects have been integrated out. Bayesian inference requires even more marginalization. In order to show that inference is not affected by allele coding, it is sufficient to show that the likelihood after integrating out the general mean is the same irrespective of allele coding. The following derivation makes no assumptions on the prior densities of marker effects. Thus, the results apply to many models, including BLUP, BayesA and BayesB in [1].

The marginal likelihood for the mixed effects model is

where *p*(**y **| *μ*, **g**, ***θ ***) is the conditional density of **y**, often a Gaussian density, and ***θ ***contains all parameters in the distribution of **e**, often only the residual variance parameter . Using the transformation result (7) in Appendix A and a change of integration variable , we can write

where *p*_0 _denotes the 012 allele coding system. Hence, the marginal likelihood does not depend on allele coding, a property used in the following derivations.

The REML-likelihood is defined when **g **and **e **are multivariate Gaussian distributed, and equals

where ***η ***contains all parameters in the distribution of **g**, commonly only the genetic marker variance parameter . This likelihood is independent of allele coding, and, hence, REML parameter estimation is independent of allele coding. Note that maximum likelihood estimation is based on *L*(*μ*, ***θ***, ***η***) = ∫ *p*(**y **| *μ*, **g**, ***θ***)*p*(**g ***|**η***)*d***g **and is affected by allele coding because, in this case, the general mean is not integrated out.

BLUP estimation of marker effects **g **assumes that the variance parameters (***θ***, ***η***) are known. The conditional distribution *p*(**g ***| ***y**, ***θ***, ***η***) = *p*(**y ***| ***g**, ***θ***)*p*(**g **| ***η***)/*L*(***θ***, ***η***) is independent of allele coding. Hence, BLUP **ĝ **and associated uncertainties do not depend on the allele coding.

In Bayesian inference, the joint posterior after integrating out *μ *is

where *p*(***θ***, ***η ***) is the joint prior for the parameters, and the denominator *p*(**y**) is the integral of the numerator. All terms in the numerator are independent of allele coding, and by marginalization *p*(**y**) satisfies the same. Hence, *p*(**g**, ***θ***, ***η **| ***y**) does not depend on allele coding.

The general intercept *μ *is, however, not independent of allele coding. For simplicity of the argument, we assume that parameters (***θ***, ***η***) are known, and omit showing these values. According to the transformation result (8) in Appendix A and a change of integration variable , the conditional expectation of the general mean is(2)

where *p*_0 _denotes density for the 012 allele coding, and  is the conditional expectation of the general mean when using the 012 allele coding. Thus, the general mean estimate is different by allele coding when  is not zero. When **g **and **e **are multivariate Gaussian distributed, the conditional expectations **ĝ **and  equal the BLUP and BLUE estimates, respectively.

Finally, the inference is indifferent to the allele being counted. This is demonstrated by studying the centered coding system and assuming that allele in the first marker is counted in the opposite way, i.e., the first column in **Z **is minus the first column in **Z***_c_*, or **z**_1 _= -**z**_*c*1_. We see that  where the entries in  are equal to the entries in **g**, except for the first entry which equals minus the first entry in **g**. Since **g **and  have the same distribution, these two models are equivalent.

### Genomic breeding values

#### Estimating breeding values

In breeding value evaluation, the main interest is in estimation of genomic breeding values for the genotyped animals. In other words, estimation of  where **ĝ **are solutions to the marker effects by a marker-based model like model (1). Because the marker effect solutions are the same for different allele coding systems, the estimated genomic breeding values are different due to differences in the coefficient matrix **Z**.

Allele coding does not, however, change relative differences between the estimated genomic breeding values, because  shows that they are just shifted by a constant. Let us define complete genomic breeding values as . Substituting  and using equation (2) we obtain

Consequently, the estimated complete breeding values  are the same irrespective of allele coding.

#### Equivalent model and allele coding

Assume that the marker effects have a Gaussian distribution  where **I***_m _*is an *m *× *m *identity matrix. The breeding values **a **= **Zg **can be calculated directly without estimating **ĝ **by the model [2,3]

where the breeding values have prior density of . Often the covariance matrix of the breeding values is scaled by a value such as  where *p_i _*is the allele frequency of marker *i*. Then, the breeding values have a prior density of  where the genomic relationship matrix is  and genetic variance is . Assuming that the residual distribution is **e **~ *N *(**0**, **R**), then the mixed model equations for the equivalent model are(3)

The breeding value solutions **â **from these mixed model equations (3) are the same as the genomic breeding values calculated by  where  are marker effects estimated by the marker-based model (1).

Therefore, the conclusion for the marker-based models about relative differences between genomic breeding values being unaffected by allele coding is also true for the equivalent model, although different allele coding methods lead to different genomic relationship matrices. Similarly, variance component estimation by REML and Bayesian methods are unaffected by the allele coding due to equivalence of models with the marker-based models.

The mixed model equations in (3) are not well-defined when the genomic relationship matrix **G **is singular. However, mixed model equations not requiring an invertible **G **matrix do exist; see page 48 in [5]. The genomic relationship matrix **G **can be singular for several reasons. For example, there can be identical twins or clones that have the same genotypes. In addition, for the centered allele coding system the genomic relationship matrix is . The last row of  is equal to the sum of all the other rows. Hence, **Z***_c _*is not of full rank, and **G***_c _*is singular.

#### Prediction error variances and reliabilities

Gaussian models are often used in practical genomic evaluation of animals. In these models, reliabilities of estimated breeding values **â **are calculated using elements of the inverse of the mixed model equations such as (3). Reliability of **â***_i _*is(4)

where PEV*_i _*is the prediction error variance, i.e., Var(*a_i _*| **y**), of animal *i*, and *G_ii _*is the diagonal element of animal *i *in the genomic relationship matrix **G**; e.g. [6], p. 51 in [7]. The prediction error variance for animal *i *is the diagonal element of the inverse of the coefficient matrix of mixed model equations (3) for animal *i*. Alternatively,(5)

where **C***^g ^*is the genomic marker effect submatrix in the inverse of the coefficient matrix of the mixed model equation for marker-based model (1) (see Appendix B). The submatrix **C***^g ^*= Var(**g **| **y**) is the same irrespective of the allele coding method used as shown in the chapter on inference on marker-based models. Because the coefficient matrix **Z **is different depending on allele coding, PEV is also different depending on allele coding. Consequently, the reliability of **â **depends on allele coding.

More generally, for any of the models considered in this paper, **a **= **Zg **where *p*(**g **| **y**) is independent of allele coding and **Z **depends on allele coding. Therefore, the distribution *p*(**a ***| ***y**) and, in particular, the variance-covariance matrix Var(**a **| **y**) and reliabilities of **â **depend on allele coding.

The complete breeding value distribution *p*(**a***_d _| ***y**) does not depend on allele coding, unlike PEV associated with **â**. The proof is based on the demonstration that when applying any function *f*, the expectations are independent of the allele coding system,

where E_0 _is the expectation when using the basic allele coding method. Therefore, the variance-covariance matrix Var(**a***_d _*| **y**) and all higher order moments of the distribution are independent of allele coding. However, the result does not provide actual formulas for the moments.

A closed form formula of the variance-covariance matrix is derived for a Gaussian model. Assume  and **e **~ *N *(**0**, **R**). For this model, in Appendix B we obtain that

where  and . As demonstrated earlier in this section, this variance-covariance matrix is independent of allele coding. When , the variance-covariance matrix simplifies to(6)

where **Z***_c _*is based on the centered allele coding method. The diagonal elements in (6) are different from the PEV*_i_*s in (4) because they contain uncertainty about the unknown mean *μ *as well.

For the complete breeding values **a***_d_*, we have shown above that prediction error variances are independent of allele coding and we have provided a formula for the Gaussian model. Reliabilities of **â***_d_*, however, can not be defined in a meaningful way. Substituting the diagonal elements from (6) for the PEV*_i_*s in (4) is not appropriate since the denominator in (4) is Var(**a***_i_*) not Var(**a***_d_*)*_ii_*. The denominator in the reliability formula should contain the marginal (unconditional) variance Var(**a***_d_*)*_ii _*= *∫ *Var(*μ *+ **a***_i _*| *μ*)*dμ*, but this variance is infinite.

### McMC computations

#### Theoretical convergence rate

The convergence and mixing of an McMC algorithm depend on the parametrization of the model and on the algorithm used. Theoretical results about the geometric rate of convergence to the stationary distribution for Gibbs sampling algorithms are shown in [8], and as mentioned in [9] this rate also describes the mixing of the algorithm. Below we show specific results about the convergence rate *ρ *for Gibbs sampling algorithms for simulating from [*μ*, **g **| **y**] in the marker-based model (1), where **g **is Gaussian  and **e **is Gaussian . These results provide some ideas about more general models and algorithms where theoretical results cannot be obtained.

Section 2.2 in [8] contains results about various Gibbs-sampling schemes for simulating from a multivariate distribution. Here we apply these results (see Appendix C for details) to two types of Gibbs updating schemes. The first scheme iterates between updating *μ *and a block of all components in **g**, and will be called the block updating scheme hereinafter. The second scheme updates *μ*, *g*_1_, ..., *g_m _*sequentially one at a time, and will be called the single site updating scheme.

For the block scheme, the convergence rate is

where  is a *m *× 1 vector, and .

For the single site scheme, the convergence rate is

where **L***_g _*is the matrix containing the lower triangle and the diagonal of **D**^-1 ^**C***_g_*, **D **is the diagonal of **C***_g_*, and **U***_g _*is the matrix containing the upper triangle of **D**^-1 ^**C***_g_*. This single site Gibbs sampling algorithm is the stochastic counterpart of the Gauss-Seidel algorithm for solving the mixed model equations, and the convergence results are similar, see [10].

When the centered allele coding method  is used,  is a vector of zeros and, hence, *ρ*_1 _= 0. The centered allele coding method breaks dependency between the general mean and genetic marker effects, as seen from the variance-covariance matrix (derived in Appendix B for a more general situation)

Consequently, absorption of the general mean is done without needing to compute absorption explicitly. Note that, in general, this holds only when the residual variance-covariance matrix is . For the block McMC scheme, the convergence and mixing of the algorithms are of the same order as for non-McMC algorithms that simulate directly from the distribution of interest. For the single site McMC scheme, the centered allele coding method still breaks the dependence between the general mean and marker effects, but as *ρ*_2 _*>*0 illustrates, the individual marker effects *g*_1 _, ..., *g_m _*are not independent and the McMC samples are autocorrelated.

### Data and methods

#### Data

Data for the XII*^th ^*QTLMAS workshop [11] were used to illustrate the theory. The simulated data had four generations. In each generation, 15 sires and 150 dams were selected randomly to produce the next generation. Each sire was mated to 10 dams and each mating produced 10 progeny. Thus, the base generation had 165 individuals, and the subsequent three generations, 1500 individuals each. In total, the analyzed data had 4665 animals with phenotypes. The simulated trait had a heritability of 0.30. The data had 6000 equally spaced SNP markers on six chromosomes. We deleted markers that had a minor allele frequency less than 1% among the phenotyped individuals and this reduced the number of markers to 5896.

The 012 allele coding method was used to make the base data set. So, the least frequent allele was counted. In addition, 210, 101, and centered allele coding data sets were analyzed.

#### Variance component analysis

The marker-based model (1) with common genetic variance was used to analyze the data:  and . McMC computations by a single site updating Gibbs sampler were used to calculate posterior mean estimates of the location (*μ*, **g**) and dispersion  parameters. The length of the McMC chain was 100000 iterations, of which the burn-in period of 10000 was omitted. Every tenth sample was saved, giving 9000 saved samples. Effective sample sizes were calculated for all parameters using the initial monotone sequence approach [12]. The approach estimates the number of independent samples from the post burn-in samples.

Theoretical convergence rates were calculated for all the allele coding methods in the single site and block updating schemes. Here, the convergence rate also describes the mixing of the McMC chain and was measured by the correlation between successive McMC samples. Because of the Markov property, the *k*-lag correlation is *ρ^k^*, where *ρ *is the convergence rate and *k *is the lag or distance between samples. In order to compare the theoretical convergence rate to the observed effective sample size, theoretical mixing was calculated relative to the 012 allele coding system as follows. Let *ρ*_012 _be the convergence rate from the 012 allele coding system, and *ρ *the convergence rates from another allele coding system. Mixing of the other allele coding system is equal to mixing of the 012 allele coding system when every *k^th ^*sample is taken from the McMC samples and *ρ*_012 _= *ρ^k^*. Thus, the relative mixing is *k *= log(*ρ*_012_)*/ *log(*ρ*).

Parameter estimation by REML for both the marker-based model and the equivalent model and for all four allele coding methods was done using software DMU [13]. Both AI-REML and EM-REML were investigated. For the equivalent model and the centered allele coding method, the singular genomic relationship matrix was modified by multiplying the diagonals by 1.001 in order to be able invert the matrix. The effect of allele coding on convergence was investigated, and it was checked that the parameter estimates were the same.

#### Reliabilities of genomic breeding values

Reliabilities were calculated by different allele coding methods using (4) and elements of the inverse of the mixed model equations. The variance components were those estimated by the centered allele coding method using the single site McMC approach. Prediction error variances were calculated by both the marker-based model equations (5) and equivalent model equations (3). Mean, minimum, maximum and standard deviations of reliabilities were calculated for all allele coding methods. Also, correlations between reliabilities from all allele coding methods were calculated.

## Results and Discussion

Posterior mean estimates of marker effects and variance components were almost equal between different allele coding methods (Table 1). Correlations of estimates of the marker effects between allele coding methods were higher than 99.98%. Only the general mean (*μ*) had a different estimate, as expected. The estimated variance components agreed well with those used to simulate the data. Additive genetic variance was , where , and *p_i _*are the observed allele frequencies in the reference data. Thus, the heritability estimate was , compared to the simulated value of 0.30.

**Table 1:** Posterior means of selected parameters by allele coding

	Allele coding			
**Parameter**	**012**	**210**	**101**	**centered**

*μ*	1.698	0.801	1.083	1.359
	6.700 × 10^-4^	6.703 × 10^-4^	6.691 × 10 ^-4^	6.698 × 10^-4^
	2.996	2.996	2.996	2.996

Effective sample sizes differed depending on allele coding method. The centered allele coding method had the best mixing, and the 210 allele coding method had the worst (Table 2). In particular, the increase in effective sample size was largest for the general mean. For the centered allele coding method, the general mean was independent from the marker effect **g**, which led to excellent mixing of this parameter. In general, the marker effects showed excellent mixing. With all allele coding methods, effective sample sizes were at least 5500 for all marker effects, and on average were equal to about 8800.

**Table 2:** Effective sample sizes in McMC computations by allele coding

	Allele coding			
**Parameter**	**012**	**210**	**101**	**centered**

*μ*	46	9	96	8961
	723	330	1001	1701
	7814	6861	7661	7720

Theoretical convergence rates (Table 3) displayed the same results as the effective sample sizes discussed above. Note that our Gibbs sampler used single site updates for all parameters. For the single site updating algorithm, the 210 allele coding system was predicted to need 5.64 times more iterations than the 012 allele coding system. The number of effective samples for the general mean parameter (*μ*) was 5.11 times bigger for the 012 than for the 210 allele coding system. These figures were 0.48 and 0.48 for the 101 allele coding system, and 0.070 and 0.0051 for the centered allele coding system. Theoretical convergence rates for the block Gibbs sampler showed the same pattern as for the single site update (Table 3).

**Table 3:** Predicted absolute and relative convergence rates in McMC computations by allele coding

	Allele coding				
		**012**	**210**	**101**	**centered**

single site	*ρ*_2_	0.9974795	0.9995523	0.9947515	0.9647670
	relative	1.00	5.64	0.48	0.070
block	*ρ*_1_	0.9995429	0.9998860	0.9989434	0.00
	relative	1.00	4.01	0.43	-

Surprisingly, the block Gibbs sampler was predicted to be worse than the single site Gibbs sampler for all allele coding systems except for the centered allele coding system. However, it is well known in the literature that block-updating schemes may sometimes be worse than single site updating schemes, for examples see [8]. The excellent convergence rate of the block Gibbs sampler with centered allele coding was expected because, in this case, the Gibbs sampler is equal to Monte Carlo sampling.

Table 4 shows convergence of REML in parameter estimation. For the marker-based model, the convergence was independent of the allele coding system, whereas for the equivalent model, the convergence was fastest for the centered coding system and slowest for the 210 coding system, although the differences were small. The parameter estimates obtained (Table 5) were the same, with the exception of  for the centered coding system. The difference is due to the need to make the genomic relationship matrix **G***_c _*to be full rank by multiplication of the diagonals by 1.001. In summary, REML parameter estimation is only slightly affected by allele coding.

**Table 4:** Number of iterations in REML by allele coding and model

Model	Allele coding	AI-REML	EM-REML
Marker	012	9	45
Marker	210	9	45
Marker	101	9	45
Marker	centered	9	45

G	012	7	34
G	210	9	44
G	101	7	31
G	centered	7	28

**Table 5:** REML estimates by allele coding

	Allele coding				
**Model**	**Parameter**	**012**	**210**	**101**	**centered**

Marker		6.623 × 10^-4^	6.623 × 10^-4^	6.623 × 10^-4^	6.623 × 10^-4^
Marker		2.993	2.993	2.993	2.993

G		1.540	1.540	1.540	1.540
G		2.993	2.993	2.993	2.992

Reliabilities were affected depending on the allele coding method used. Differences were large (Table 6). Average reliabilities ranged from 0.37 with the 210 allele coding method to 0.80 with the centered allele coding method. The centered allele coding method gave higher reliabilities than achieved by any of the other allele coding methods. Reliabilities calculated by different allele coding methods were also different as judged by the correlation to each other (Table 7). Reliabilities calculated by the marker-based model and the equivalent model approaches were equal within the numerical rounding error.

**Table 6:** Summary statistics of genomic breeding value reliabilities by allele coding

Allele coding	min	mean	max	std
012	0.41	0.49	0.59	0.022
210	0.30	0.37	0.42	0.017
101	0.55	0.62	0.73	0.024
centered	0.72	0.80	0.95	0.026

**Table 7:** Correlations between genomic breeding value reliabilities by allele coding

Allele coding	210	101	centered
012	-0.22	0.32	0.43
210		0.82	0.59
101			0.91

The observed large differences in reliabilities using different allele coding methods can be explained by differences in estimation uncertainty. Different allele coding systems have different **Z **matrices. Consider first the 012 and 210 allele coding methods. The 012 allele coding system has a 0 coefficient when the individual is homozygous for the more frequent allele while the 210 allele coding system has a coefficient of 2 instead. In the marker-based model, uncertainty or the inverse of the coefficient matrix is the same irrespective of allele coding method. Reliability is calculated by multiplying the marker uncertainty by the **Z **matrix (5). Consequently, uncertainty is less in the 012 allele coding system than in the 210 allele coding system because the more frequent homozygous allele multiplies the marker solution and uncertainty by zero. Thus, homozygous genotypes for the more frequent allele do not increase uncertainty when estimating genomic breeding values. Thus, the 012 allele coding system will yield higher reliabilities than the 210 allele coding system, as was observed. This argument can be generalized as follows. In the genomic model considered, uncertainty of a genotype in estimating genomic breeding value is valued relative to a chosen base genotype. The further away an observed genotype is from the base genotype, the larger the coefficient in absolute value in the **Z **matrix and the higher the uncertainty in genomic breeding value. In the 012 allele coding system, the base genotype is homozygous for the more frequent allele, while in the 210 allele coding, it is homozygous for the less frequent allele. In the 101 allele coding system the base genotype is the heterozygote. The higher the number of heterozygous individuals is in the data the smaller will the uncertainty be, i.e., the higher the reliability will be for the 101 allele coding system. For the centered allele coding system the base genotype is the average genotype in the data. Thus, for this allele coding system the base population is roughly the population we work with [14], and it has the smallest average distance of observed genotypes from the base genotype. In practice, this can be expected to lead to the highest reliabilities.

Different allele coding systems have different model design matrices **Z**, and, hence, imply different models. Thus, reliabilities from different allele coding systems are in fact from different statistical models. Comparison of reliabilities from different models is meaningless. However, the different allele coding systems lead to the same parameter estimates. If the correct allele coding method, i.e., statistical model, is known, it should be used. Because the true model is unknown and comparison of reliabilities by allele coding method is meaningless, some principles must be used to decide on which allele coding method should be used. These principles will not guarantee the use of a correct model or correct reliabilities. One such principle should be consistency of reliabilities between evaluations. The centered allele coding method changes model from one evaluation to the next because more marker data accumulate. Hence, according to the consistency principle, it cannot be recommended to computate reliabilities. Likewise, the base genotype in the 012 and 210 allele coding methods depend also on the observed allele frequencies, i.e., marker data.

The centered allele coding method is similar to that introduced in [4] where the allele frequencies were from an unselected base population. It was used in order to "give more credit to rare alleles than to common alleles when calculating genomic relationships". As shown, inference is the same irrespective of the allele coding method when a fixed general mean is in the model. However, reliabilities are affected as shown. The use of base population allele frequencies in the centered allele coding method would remove the above mentioned problem of inconsistency between evaluations, but estimating these allele frequencies is elusive. Recently, [15] presented a method for adjusting the **G***_c _*relationship matrix to become a relationship matrix relative to the base population, thereby avoiding the estimation of base population allele frequencies.

The results in this paper are based on the assumption that phenotypes and genotypes are available for all animals in the analysis. This assumption may often not be satisfied. Models based on an extension of the genomic relationship matrix to include also non-genotyped animals have been presented by [16-18]. The results in the present paper about parameter estimates and estimated breeding values not depending on allele coding do not carry over to the models with an extended genomic relationship matrix.

## Conclusions

We showed that, in theory, different allele coding methods led to the same inference in marker-based models when the model has a fixed general mean effect. Practical analyses led to the same conclusions. Also in theory, the centered allele coding method was expected to give better mixing properties when Markov chain Monte Carlo methods were used. This was also observed in practice. When an equivalent breeding value model was used, different allele coding methods proved to lead to the same inference as in the marker-based model. However, reliabilities of breeding values depend on the chosen allele coding system because different allele coding methods change the amount of uncertainty in the estimated breeding values.

## Appendix A

In the following, we consider the effect of allele coding method on the densities *p*(**y **| *μ*, **g**) and *p*(*μ*, **g**, | **y**). For simplicity of presentation, the parameters in the distribution of **g **and **e **are omitted. Let *p*_0 _denote density for 012 allele coding. Because the location parameters *μ *and marker effects **g **relate to the observations **y **only through **1***_n_μ *+ **Zg**, we first study this term. By substituting  into the term, we have

So, when different allele coding systems are used, the densities have equality by(7)

By changing the integration variable , we obtain ∫ *p *(**y **| *μ*, **g**) *dμ *= ∫ *p_0 _*(**y **| *μ_0_*, **g**) *dμ_0 _*and, hence, *p*(**y**) = ∫ ∫ *p*(**y **| *μ*, **g**)*p*(**g**)*dμd***g **= *p*_0_(**y**). From these results, we see that(8)

The results (7) and (8) are fairly general in terms of distributional assumptions. The only requirements are that *p*(**y **| *μ*, **g**) depends on *μ *and **g **only through **1***_n_μ *+ **Zg**, that an improper uniform prior is used for *μ*, and that *p*(**y**) is finite. The later requirement is to assure the posterior distribution becomes a proper distribution, and this has to be proven for a model to be valid when an improper prior is used.

When **e **~ *N*(**0**, **R**), it is not difficult to show that

with . Therefore, ∫ *p *(**y **| *μ*, **g**) *dμ < c*_0 _where the constant *c*_0 _is independent of **g**. Thus, *p*(**y**) = ∫ ∫ *p*(**y **| *μ*, **g**)*dμp*(**g**)*d***g ***< c*_0 _∫ *p*(**g**)*d***g **= *c*_0 _is finite, irrespective of distribution of the marker effects **g**.

## Appendix B

We consider a Gaussian distribution model where  and **e **~ *N *(**0**, **R**). Consequently, the distribution [*μ*, **g **| **y**] is a multivariate Gaussian distribution. In the following, we derive the variance-covariance matrix for this distribution. The conditional density is

where

With ,  and . The matrix **Q **is the coefficient matrix in the mixed model equations and the inverse of this matrix is

where . Note that the submatrix **C***^g ^*= Var(**g **| **y**) is independent of allele coding, because, as shown in the main text, *p*(**g **| **y**) does not depend on allele coding.

Variance-covariance matrix for the complete breeding value is

## Appendix C

The results in [8] state that the convergence rate of a Gibbs sampler is equal to the largest modulus eigenvalue of a certain matrix **B **where the eigenvalues can be complex numbers. As mentioned in [9] this convergence rate is also a a measure of correlation between successive McMC samples, i.e., mixing of the algorithm. The closer the convergence rate is to zero the less correlated are the successive samples.

The **B **matrix is constructed as follows. Let **Q **be the inverse of the variance-covariance matrix of the target multivariate normal distribution, in our case the coefficient matrix in the mixed model equations. Assume that a Gibbs sampling scheme is used where the variables are grouped into *s *blocks and **Q **is split accordingly into *s *blocks. First, define

Let **L **be the block lower triangular matrix with blocks in the lower diagonal being those of **A**, and let **U **= **A **- **L**. Thus, **U **is a strictly upper triangle matrix with zeros in the diagonal. Then the matrix of interest is

We consider the genomic marker model (1) where **g **is Gaussian  and **e **is Gaussian . The conditional distribution [*μ*, **g **| **y**] is a multivariate normal distribution with some mean vector and a variance-covariance matrix with inverse

where ; see Appendix B. We consider two McMC updating schemes. The first scheme iterates two blocks: *μ *and **g**. The second scheme updates successively all parameters: *μ*, *g*_1_, ..., *g_m_*. For the first McMC updating scheme we have

where  is a *m *× 1 vector. Hence,

The convergence rate is

where *ρ_lv_*(**B**) of a matrix **B **is a notation for the maximum modulus eigenvalue of **B**. The final equality follows from a general property for a square matrix form **Cvv' **where **v **is a vector, saying that it only has one eigenvalue different from zero which is equal to **v**'**Cv**.

For the second McMC update scheme,

where **D **is the diagonal of **C***_g_*. Hence,

where **U***_g _*is an upper triangular matrix containing the upper triangle of **D**^-1 ^**C***_g _*and **L***_g _*is a matrix containing the diagonal and lower triangle of **D**^-1 ^**C***_g_*. Therefore,

The convergence rate is

## Competing interests

The authors declare that they have no competing interests.

## Authors' contributions

IS wrote the first drafts of the manuscript and OFC helped to revise and finalize it. IS and OFC derived the formulae together. IS did all the data analysis except the REML computations which were done by OFC.
